# Therapeutic opportunities for hypermutated urothelial carcinomas beyond immunotherapy

**DOI:** 10.18632/oncoscience.596

**Published:** 2024-04-25

**Authors:** Ioannis A. Voutsadakis

**Keywords:** bladder cancer, transitional cell carcinoma, immune checkpoint inhibitors, targeted therapy

The tumor mutation burden (TMB) is a novel clinical biomarker for prediction of checkpoint inhibitor immunotherapy response across cancers and high TMB has been used as a tumor agnostic indication for treatment with the PD-1 inhibitor pembrolizumab [1]. High TMB is also associated with defects in mismatch repair (MMR) proteins producing the microsatellite instability (MSI) phenotype, which is also a biomarker of response to immune checkpoint inhibitors [2]. However, both biomarkers are imperfect and not all cancers with high TMB or MSI phenotype respond to immunotherapy. The reason for this phenomenon may relate to additional alterations present in some tumors with high TMB or may be due to differences in the immune environment of diverse cancers [3]. Conversely, some tumors with no MMR alterations have high TMB, and their hypermutability, which is due to other defects, such as pathogenic proofreading polymerase epsilon (POLE) mutations, may still lead to immunotherapy sensitivity [4].

A sub-set of urothelial carcinomas possess a high TMB. With a cut-off of 10 mutations/Mb, about one in four urothelial bladder carcinomas has a high TMB in the urothelial cancer series of TCGA [5]. Urothelial carcinomas with high TMB have only rarely MMR protein or POLE mutations but present additional alterations in higher frequency than cancers with low TMB, including mutations in several epigenetic modifiers [5]. Genes encoding for histone methyltransferases KMT2C and KMT2D, acetyltransferase p300 and the member of SWI/ SNF complex ARID1A are mutated in 25% to 45% of urothelial cancers with high TMB [6]. Moreover, several genes encoding for proteins of the DNA damage response (DDR) are mutated in a significant proportion of these cancers, including BRCA2 (22.6%), ATM (25.5%) and POLQ (21.7%). DDR following double strand breaks is particularly active in areas of the genome with high transcriptional activity, where the epigenetic machinery associated with transcription is also a key player that unwinds chromatin, dislocates or removes histone octamers and provides the epigenetic marks for recruitment of transcription factors and the general transcription machinery [7]. ARID1A mutations are associated with MMR defects and with response to immune checkpoint inhibitors [8]. DDR defects potentiated by PARP inhibitors promote activated T lymphocyte tumor infiltration and cytotoxicity. Therefore, alterations in both DDR and epigenetic modifiers may work in tandem to create a high TMB. Both DDR defects and epigenetic modifier defects may be targetable with currently available drugs such as PARP inhibitors and BET inhibitors [9].

The underlying mechanisms creating mutations are not only important as therapeutic targets but are also of significance due to the different mutational signatures that they produce. Urothelial cancers with high TMB possess more frequently COSMIC mutation signatures SBS2 and SBS13, in which a TC dinucleotide becomes TT or TG, as opposed to cases with low TMB, where signature SBS5 is more prevalent [6]. Neo-antigens created with these specific signatures may have different ability to be presented by the antigen presentation machinery compared to other neo-antigens, leading to differential immunogenicity and sensitivity to checkpoint inhibitors. In addition specific alterations frequently co-exist, creating opportunities for co-targeting. As an example *BRCA1* mutations frequently happen in cases with *TP53* mutations and could be targeted with PARP inhibitors associated with a TP53 neo-antigen targeted vaccine, augmented by a checkpoint inhibitor [10].

Besides mutations, copy number alterations have a different prevalence in urothelial cancers with high and low TMB. Of interest, *NECTIN4* amplifications are more frequent in high TMB cancers (27.1% of cases in TCGA) than in low TMB tumors (13.6% of cases in TCGA). In a recently completed phase 3 clinical trial, a monoclonal antibody drug conjugate, enfortumab vedotin, targeting nectin 4, in combination with pembrolizumab was more effective than the standard platinum based doublet chemotherapy in the first line setting and prolonged significantly both PFS and OS of metastatic urothelial carcinoma patients [11]. In this trial, patients were not selected for nectin 4 expression or TMB, as some degree of nectin 4 expression is ubiquitous in most urothelial carcinomas. It would be of interest to analyze responses and survival outcomes according to nectin 4 expression or amplification of its gene. These results argue that combinations based on immunotherapy may also provide an opportunity for targeting urothelial cancers with low TMB, and provide efficacy superior to classic chemotherapy. Combinatorial approaches based on immunotherapy and targeting additional molecular defects, that are present in urothelial carcinomas, hold the hope for successful therapy of the sub-set of immune checkpoint inhibitor resistant urothelial carcinomas with high TMB and of urothelial carcinomas with low TMB.
